# Dual-modal super-resolution ultrasound and NIR-II fluorescence imaging of ischemic stroke with ICG-doped porous PLGA microspheres

**DOI:** 10.1016/j.mtbio.2025.101513

**Published:** 2025-01-22

**Authors:** Ziyue Li, Yu Qiang, Dongli Chen, Dehong Hu, Duyang Gao, Xiaohua Xu, Lei Sun, Yingjia Li, Weibao Qiu, Zonghai Sheng

**Affiliations:** a Department of Medicine Ultrasonics, Nanfang Hospital, Southern Medical University, Guangzhou, 510515, China; b Research Center for Advanced Detection Materials and Medical Imaging Devices, Paul C. Lauterbur Research Center for Biomedical Imaging, Institute of Biomedical and Health Engineering, Shenzhen Institute of Advanced Technology, Chinese Academy of Sciences, Shenzhen, 518055, China; c The Hong Kong Polytechnic University, Department of Biomedical Engineering, Hong Kong, 999077, China; d Division of Ultrasound, The University of Hong Kong-Shenzhen Hospital, No.1, Haiyuan Road, Futian District, Shenzhen, 518053, China; e Key Laboratory of Biomedical Imaging Science and System, Chinese Academy of Sciences, State Key Laboratory of Biomedical Imaging Science and System, Shenzhen, 518055, China

**Keywords:** NIR-II fluorescence, Super-resolution ultrasound, Indocyanine green, Porous microsphere, Ischemic stroke

## Abstract

Ischemic stroke, resulting from the obstruction of blood flow to the brain, remains a leading cause of morbidity and mortality worldwide. Traditional imaging modalities, such as magnetic resonance imaging and computed tomography, while effective for identifying stroke locations, are often limited in their ability to detect early pathological changes due to constraints in spatial resolution and sensitivity. This study introduces a novel dual-modal imaging approach that employs indocyanine green-doped porous poly (lactic-co-glycolic acid) (PLGA) microspheres (ICG-pPLGA MPs) for super-resolution ultrasound and near-infrared II (NIR-II) fluorescence imaging of ischemic stroke. The porous structure of ICG-pPLGA MPs enhances their stability, prolongs their circulation time, and improves ultrasound contrast compared to commercial lipid microbubbles. Additionally, the NIR-II fluorescence allows for high-resolution and noninvasive visualization of superficial vasculature. In a rat model of ischemic stroke, we demonstrate the capability of ICG-pPLGA MPs to achieve high-resolution imaging of cerebrovascular structures and functions, surpassing the imaging performance of standard diffusion-weighted imaging. Our findings underscore the potential of this dual-modal imaging technique using ICG-pPLGA MPs to accurately characterize microvascular changes during ischemic events, thus offering valuable insights for early diagnosis and therapeutic monitoring.

## Highlights

• Dual-modality imaging of super-resolution ultrasound and NIR-II fluorescence imaging.
• New contrast agent offer higher *in vivo* stability and improved imaging efficiency.
• High-resolution imaging method for early-stage stroke detection.

## Graphical abstract

**Figure:**
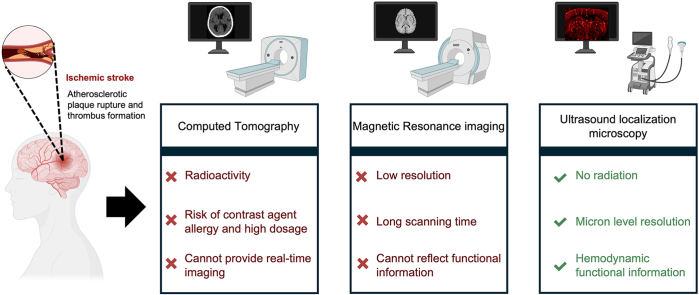

## 1 Introduction

**Scheme 1:**
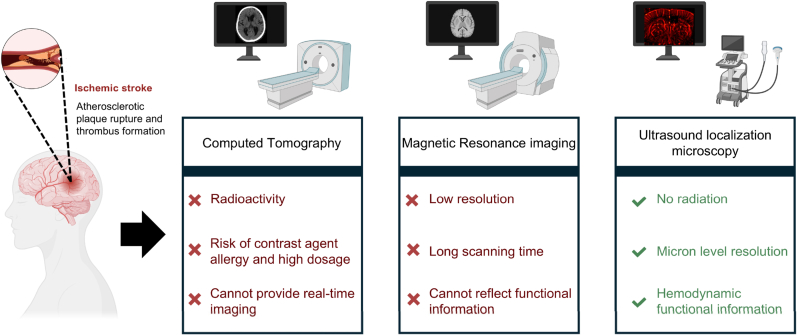
Schematic illustration of ischemic stroke and its diagnostic techniques, including computed tomography (CT), magnetic resonance imaging (MRI) and ultrasound localization microscopy (ULM). The dashed area of the brain shows the brain with an obstructed blood vessel.

Ischemic stroke, also referred to as cerebral infarction, is a neurological disorder caused by the obstruction of blood vessels in the brain [[1], [2], [3]]. This condition is characterized by a high incidence rate, significant disability, and elevated mortality, thus placing a substantial burden on patients, families, and society [4,5]. Current clinical diagnosis of ischemic stroke primarily relies on magnetic resonance imaging (MRI) and computed tomography [[6], [7], [8]]. While the imaging modalities are effective in delineating location and extent of lesions, they are limited in their ability to visualize early pathological changes due to insufficient spatial resolution (typically several millimeters) and low sensitivity in detection (typically millimolar range) [[9], [10], [11]]. Recently, emerging optical imaging techniques, such as near-infrared (NIR) fluorescence imaging [[12], [13], [14]] and photoacoustic imaging [15,16], have been developed for ischemic stroke research. These techniques offer several advantages, including high spatial resolution, superior detection sensitivity, and lower equipment costs [[17], [18], [19]]. However, the clinical application of optical imaging for stroke diagnosis remains hindered by limitations such as shallow tissue penetration depth (<1 cm) and challenges in quantitative analysis [[20], [21], [22]]. Consequently, the development of novel imaging methods that combine high sensitivity, high resolution, and the ability to penetrate deeper tissue is both necessary and urgent for the effective diagnosis and management of ischemic stroke (see Scheme 1).

In contrast to optical imaging, ultrasound imaging is widely used in clinical practice due to its deep tissue penetration, absence of ionizing radiation, real-time imaging capabilities, and the portability of imaging devices [[23], [24], [25], [26]]. Recently, ultrasound localization microscopy (ULM) has emerged as a novel technique for high-resolution visualization of vascular structures and functions [[27], [28], [29]]. ULM achieves quantitative imaging of microvessels by localizing and tracking the motion of individual microbubbles [30,31]. This technique has already found applications in early diagnosis and therapeutic monitoring across various medical disciplines, including neurology [32,33], oncology [34], nephrology [35,36] and cardiology [28,37]. However, the ULM technique requires the continuous injection of microbubbles to compensate for signal loss caused by microbubble rupture during circulation, which complicates the imaging procedure and increases the risk of adverse reactions [[38], [39], [40]]. Furthermore, super-resolution ultrasound imaging of superficial cerebral vessels can be significantly hindered by signal interference from the scalp and skull, limiting the achievable spatial resolution compared to optical imaging [[41], [42], [43]]. To date, single-modality super-resolution ultrasound imaging has not been applied in the diagnostic study of ischemic stroke.

In this study, we developed indocyanine green-doped porous poly (lactic-co-glycolic acid) (PLGA) microspheres (ICG-pPLGA MPs) for use in super-resolution ultrasound and fluorescence imaging within the second NIR biowindow (1000–1700 nm, NIR-II). Compared to conventional ultrasound lipid microbubbles, ICG-pPLGA MPs offer two advantages: (1) their rigid structure enhances stability in the bloodstream, allowing prolonged imaging without the need for continuous microbubble injections, and (2) the combined benefits of ultrasound super-resolution and NIR-II fluorescence imaging enable comprehensive visualization of both deep cerebral vasculature and high-resolution superficial vascular structures. Using ICG-pPLGA MPs in a rat model of ischemic stroke, we achieved high-resolution imaging of blood flow, demonstrating superior performance compared to clinical MRI. These findings introduce new imaging methods and technologies that enhance ULM techniques, offering a more precise approach to the diagnosis of ischemic stroke.

## 2 Materials and methods

### 2.1 Materials

Polylactic acid-hydroxy acetic acid copolymer (PLGA), distearoylphosphatidylcholine (DSPC), and ammonium bicarbonate were obtained from Yuanye Biotechnology Co., Ltd. (Shanghai, China). Indocyanine green (ICG) and agar were sourced from Aladdin Biochemical Technology Co., Ltd. (Shanghai, China). Polyvinyl alcohol (PVA) was acquired from Sigma Aldrich Trading Co., Ltd. (Shanghai, China). Perfluoro propane (C_3_F_8_ gas) was purchased from Shenzhen Hongzhou Industrial Gas Co., Ltd.

### 2.2 Preparation of ICG-pPLGA MPs

Initially, 4 g of polyvinyl alcohol (PVA) powder was weighed and dissolved in 100 mL of ultrapure water to create a 4 % PVA solution. The mixture was stirred until homogeneous and subsequently stored at 4 °C. Next, 50 mg of polylactic acid-hydroxy acetic acid copolymer (PLGA) and 2.5 mg of distearoylphosphatidylcholine (DSPC) were added to a brown vial. Following this, 1 mL of dichloromethane and 500 μg of indocyanine green (ICG) were introduced. The mixture was allowed to dissolve in a freezer for a minimum of 2 h. Subsequently, 200 μL of ammonium bicarbonate solution (60 mg/mL) was added to the PLGA/DSPC solution, and the mixture was sonicated for 2 min to obtain primary emulsion (W_1_/O). Following this, 5 mL of the 4 % PVA solution was incorporated into a primary emulsion, and the resulting mixture was homogenized for 5 min using a high-speed dispersing homogenizer while maintaining an ice bath. This process resulted in the formation of a re-emulsion (W_1_/O/W_2_). The mixture was centrifuged for 5 min (5000 rpm). After discarding the supernatant, the pellet was washed three times with ultrapure water, resulting in a green precipitate. The precipitate was lyophilized for 48 h, and the resulting powder was stored at 4 °C. The desired amount of powder was weighed and aliquoted into brown vials. These vials were subjected to a vacuum for 20 min, followed by the use of a bespoke gas exchange apparatus to replace the atmosphere with C_3_F_8_ gas. Before use, the powder was dissolved in phosphate-buffered saline (PBS) buffer. Experimental procedures were conducted in a well-ventilated area, and the experimenters wore protective masks.

### 2.3 Characterization of ICG-pPLGA MPs

The morphology of indocyanine green-doped porous PLGA microspheres (ICG-pPLGA MPs) was analyzed using field emission scanning electron microscopy (SEM) with a Zeiss Supra55 instrument (Carl Zeiss, Germany). The hydrodynamic diameters and zeta potentials of the ICG-pPLGA MPs were measured using a dynamic light scattering (DLS) instrument (Malvern, UK). The absorption, near-infrared I (NIR-I), and near-infrared II (NIR-II) fluorescence emission spectra of the ICG-pPLGA MPs, free ICG, and PLGA microspheres were assessed using a UV–Vis spectrophotometer (PerkinElmer Lambda 25), a fluorescence spectrometer (Edinburgh Instruments FLS900), and a steady-state/transient fluorescence spectrometer (Life Spec-PsFSP920), respectively. These characterization techniques provided comprehensive insights into the structural and optical properties of the ICG-pPLGA MPs, which are essential for their potential applications in imaging. Molar extinction coefficient calculation: Dilute ICG and ICG-pPLGA MPs with gradient concentration, measure their absorbance using a UV visible spectrophotometer, and plot a standard curve with solution concentration as the x-axis and absorbance as the y-axis. According to the Beer Lambert law A = *εcl*, the molar extinction coefficient *ε* is the slope of the curve, measured in L · mol ⁻^1^ cm ⁻^1^, where *l* is the optical path length. Quantum yields calculation: Prepare free ICG and ICG-pPLGA MPs solutions with five different absorption values below 0.1 at 808 nm. Use 808 nm laser as the excitation source and 1000 nm long pass filter as the emission filter to record the emission of these solutions between 1000 and 1650 nm. Then determine the integrated NIR-II area as a function of the absorption value at 808 nm.

### 2.4 *In vitro* ultrasound imaging

#### 2.4.1 *In vitro* ultrasound contrast imaging of ICG-pPLGA MPs

The *in vitro* contrast imaging effects of ICG-doped PLGA microspheres (ICG-pPLGA MPs) were evaluated using a linear array probe in conjunction with a Mindray color Doppler ultrasound diagnostic system. The mechanical index (MI) was set to 0.85, and the contrast mode was selected. ICG-pPLGA MPs and SonoVue were prepared at various concentration gradients (n = 3) of 1 × 10^5^/mL, 1 × 10^6^/mL, 1 × 10^7^/mL, 1 × 10^8^/mL, and 1 × 10^9^/mL, and were subsequently introduced into the pores of a 2 % agarose phantom for imaging observations. ImageJ software was employed to quantitatively analyze the stored imaging data and generate a corresponding correlation curve.

#### 2.4.2 Effects of mechanical indices on *in vitro* ultrasound contrast

Using the same commercially available Mindray color Doppler ultrasound diagnostic equipment, an L12-3 linear array probe was utilized to evaluate the contrast imaging effects at various mechanical indices: 0.062, 0.085, 0.112, 0.142, 0.189, 0.226, and 0.269 (n = 3). ICG-doped PLGA microspheres (ICG-pPLGA MPs) and SonoVue were maintained at a concentration of 1 × 10^6^/mL and introduced into the 2 % agarose phantom for imaging. ImageJ software was employed for the quantitative analysis of the imaging data, enabling the generation of a relationship curve.

#### 2.4.3 Ultrasound contrast stability of ICG-pPLGA MPs and SonoVue microbubbles

The stability of different contrast agents during *in vitro* imaging was evaluated using the Vevo2100 ultrasound imaging system equipped with an MS-250 probe, set to a frequency of 18 MHz and an energy level of 4 %. The contrast intensity of ICG-doped PLGA microspheres (ICG-pPLGA MPs) and SonoVue was initially normalized to an equivalent level. Dynamic images were acquired every 5 min for 30 s (n = 3). Quantitative analysis was performed using the VevoCQ analysis software provided with the imaging device. A change curve illustrating the relationship between time and contrast intensity was subsequently plotted to assess stability.

### 2.5 In vivo cerebral vascular super-resolution ultrasound imaging

Adult male Sprague-Dawley rats (200–250 g, SPF grade) were sourced from Zhuhai Baishitong Biotechnology Co., Ltd. Following intraperitoneal anesthesia, the fur on the tops of the rats' heads was carefully shaved. The animals were then secured in a stereotactic apparatus, and a rectangular cranial window (12 × 8 mm) was created using a dental drill. Ultrasound coupling gel was applied between the probe and the scalp to ensure optimal acoustic transmission. The ultrasound probe was positioned over the predetermined imaging site.

Intravenous injections of SonoVue and ICG-doped PLGA microsphere (PMs ICG) microbubbles, both at concentrations of 4 × 10⁸/mL, were administered at a rate of 150 μL/min for 2 min while imaging data were collected. Following the infusion, imaging data were acquired at fixed time intervals (5–7 min, 10–12 min, 15–17 min, and 20–22 min), with each imaging session lasting 2 min. This protocol enabled the assessment of cerebral vascular dynamics and the performance of the contrast agents over time.

The acquired contrast images were then processed to obtain super-resolution ultrasound images. Initially, a spatial-temporal filter was applied to the time-continuous B-mode images to differentiate the microbubble signals from the tissue signals [44]. This step aimed to minimize the interference from tissue signals on the microbubble signals. Sub-wavelength localization of the microbubble centers was performed on the filtered images by identifying the local maxima. The local maxima and their surrounding pixels were correlated with the ideal point spread function (PSF). Only those local maxima with a correlation greater than 0.65 were retained.

Subsequently, a radial symmetry algorithm was employed to localize the microbubble centers with sub-wavelength precision [45]. Once the microbubble localization was complete, microbubbles were tracked through continuous frames. The Kukn-Munkras algorithm was applied to pair microbubbles in two consecutive frames, ensuring the minimum total Euclidean distance between paired microbubbles. If microbubbles could be paired consecutively for more than 15 frames (approximately 15 ms), the pairings were retained as a continuous microbubble motion trajectory. Finally, all trajectories were accumulated and displayed to produce the super-resolution ultrasound image.

The positions, velocities, and directions of motion of the microbubbles within these trajectories provide insight into the hemodynamic characteristics, such as blood flow speed and direction within the cerebral vessels (Fig. 3A).

### 2.6 *In vivo* NIR-II fluorescence imaging of cerebrovascular

Adult C57BL/6 mice (male, 6–8 weeks, SPF grade) were obtained from Zhuhai Baishitong Biotechnology Co., Ltd. To evaluate the *in vivo* NIR-II imaging performance of ICG-doped PLGA microspheres (ICG-pPLGA MPs), mice were anesthetized via intraperitoneal injection. An intravenous injection of ICG-pPLGA MPs (2 × 10^8^/mL) was administered through the tail vein. Fluorescence images of the cerebral vasculature were acquired using a NIR-II fluorescence imaging system (NIRvana 640, Teledyne Princeton Instruments) equipped with an 808 nm excitation source and a long-wave pass filter set at 1200 nm. Imaging was conducted at several time points (0, 1, 2, 3, 4, and 5 min) post-injection to monitor the distribution and dynamics of ICG-pPLGA MPs within the cerebral blood vessels. This approach facilitated the assessment of the imaging capabilities and temporal behavior of the contrast agent in a live animal model.

### 2.7 Rat model of ischemic stroke

The autologous ischemic stroke model was established according to previously documented protocols [46]. Adult male Sprague-Dawley (SD) rats were anesthetized, and the right common carotid artery (CCA) was carefully isolated. The CCA was then placed in a YLS-14B thrombus tester (Jinan Yiyan Technology Development Co., Ltd.). A direct current of 1.00 mA was applied to the CCA for 1 min, followed by an additional 4 min to facilitate thrombus formation. The formed thrombus was then crushed into uniform fragments using custom-designed soft forceps. Upon releasing the clip, the fragments were allowed to flow into the internal carotid artery with arterial pulsation. The common carotid artery was occluded for 15 min to ensure that the thrombus was directed to the middle cerebral artery. After the procedure, the skin was stitched up, and the rats were allowed to recover from anesthesia. Neurological function was assessed to confirm the successful establishment of the model, employing the Longa score (ranging from 1 to 3 points) as a measure of infarct severity [47].

### 2.8 Diffusion-weighted imaging of the ischemic stroke

Imaging was conducted using a 9.4T small animal MRI scanner (Shanghai United Imaging Medical Technology Co., Ltd.). Following anesthesia, the rats were positioned in a prone orientation to ensure that their heads were centered within the MRI coil, facilitating coronal imaging. The diffusion-weighted imaging (DWI) sequence was configured with the following parameters: repetition time (TR) of 5000 ms, echo time (TE) of 35.5 ms, field of view (FOV) of 20 × 25 mm, slice thickness of 0.5 mm, and no slice spacing. These settings enabled the acquisition of high-resolution images for assessing ischemic regions within the brain tissue.

### 2.9 Super-resolution ultrasound imaging of ischemic stroke

Ultrasound coupling gel was applied between the probe and the scalp to enhance signal transmission. The probe was positioned at the coronal section of the hippocampus and corpus callosum (designated as β = 0) using real-time B-mode ultrasound imaging. The probe was subsequently translated along the longitudinal axis of the rat's body to collect data from four different sagittal planes (β, β+1 mm, β+2 mm, and β+3 mm). ICG-pPLGA MPs, prepared at a concentration of 4 × 10^8^/mL, were injected via the tail vein at a rate of 150 μL/min, with continuous infusion maintained for approximately 2 min while imaging data were simultaneously acquired from the four planes. MRI images corresponding to the same anatomical locations were then obtained for comparative analysis.

### 2.10 2,3,5-Triphenyltetrazolium chloride (TTC) staining analysis

Rat brain tissue was stained with 1 % TTC 24 h after the induction of the ischemic stroke model. Following euthanasia, intact brain tissue was extracted and frozen at −20 °C for 30 min. Coronal brain slices were prepared using a rat brain mold to produce slices from the frontal lobe to the dorsal hemisphere, each 1 mm thick. The slices were immersed in TTC staining solution and incubated in a 37 °C incubator for 20 min, with gentle inversion every 10 min to ensure uniform staining while protecting the samples from light. After rinsing with PBS, the sections were fixed in 4 % paraformaldehyde for 1 h. Upon completion of fixation, excess liquid was carefully removed, and the sections were photographed for documentation and analysis.

### 2.11 Biotoxicity evaluation of ICG-pPLGA MPs

b. End3 cells, representing normal cells, were seeded into 96-well plates at a density of 8 × 10³ cells per well. Subsequently, 100 μL of medium containing ICG-pPLGA MPs at concentrations ranging from 0 to 5 mg mL⁻^1^ was introduced into each well and incubated at 37 °C for 24 h. Afterward, 10 μL of CCK-8 solution was added, and the absorbance at 450 nm was measured using a microplate reader (PerkinElmer, US). Cell viability was then calculated based on the measurements, with five replicates per group.

Different concentrations of ICG-pPLGA MPs (0.5 mg/mL, 1 mg/mL, 2.5 mg/mL, 5 mg/mL, and 10 mg/mL), along with pure water (positive control) and PBS (negative control), were incubated with a 2 % red blood cell suspension at 37 °C for 4 h. After incubation, the samples were centrifuged at 9000 rpm for 5 min, and the supernatants were collected to measure the absorbance at 540 nm using a microplate reader. The hemolysis rate was calculated using the following formula:

(1) $$\text{Hemolysis}\;\text{rate}\;\left(\%\right)=\frac{\text{Absorbance}\;\text{of}\;\text{sample}\;–\;\text{Absorbance}\;\text{of}\;\text{control}}{\text{Absorbance}\;\text{of}\;\text{positive}\;\text{control}\;–\;\text{Absorbance}\;\text{of}\;\text{control}}\times 100\%$$

Healthy male C57BL/6 mice (6–8 weeks old) were divided into two groups: the control group received an intravenous injection of 200 μL saline, while the experimental group received 200 μL of ICG-pPLGA MPs solution (10 mg/mL dissolved in saline). One week post-injection, blood samples were collected for routine hematological and biochemical analyses. Subsequently, major organs, including the lungs, heart, liver, kidneys, and spleen, were harvested for examination using hematoxylin and eosin (H&E) staining.

Healthy BALB/c nude mice were utilized to investigate the biodistribution of ICG-pPLGA MPs. The ICG-pPLGA MPs were administered via tail vein injection. At predetermined time points (10 min, 30 min, 6 h, and 12 h), the major organs, including the heart, liver, spleen, lungs, and kidneys, were harvested. Fluorescence signals were captured using a small animal *in vivo* imaging system, and quantitative analysis was performed based on the fluorescence intensity and distribution patterns.

The concentration used in the toxicity assessment in this research refers to the total concentration of the sample, which includes both ICG and pPLGA MPs.

### 2.12 Statistical analysis

Data analysis was conducted using GraphPad Prism 9, with results expressed as mean ± standard deviation (SD). The significance between the two groups was assessed using Student's t-test. For multiple comparisons, statistical significance was determined using analysis of variance (ANOVA). Levels of statistical significance were indicated as ∗*P* < 0.05, ∗∗*P* < 0.01 and ∗∗∗*P* < 0.001, while "NS" denoted no statistically significant difference.

## 3 Results and discussion

### 3.1 Characterization of ICG-pPLGA MPs

**Fig. 1:**
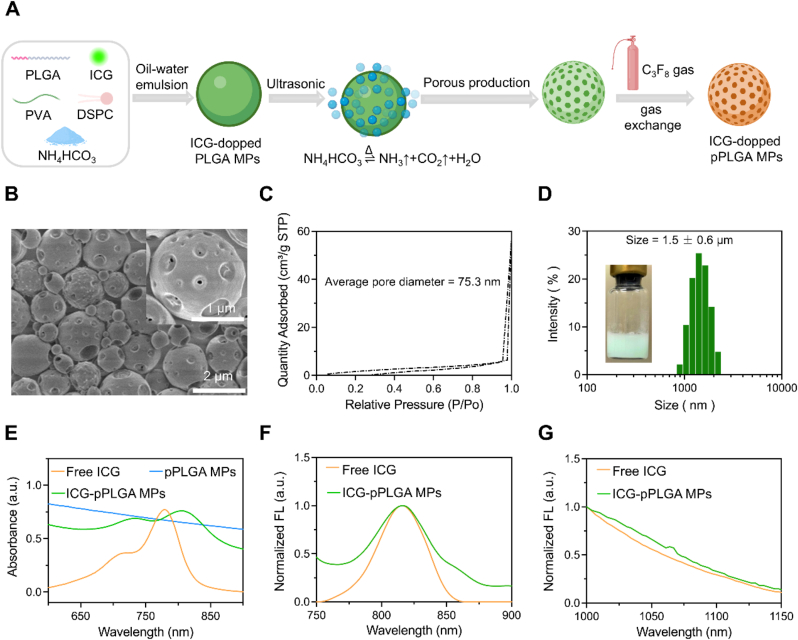
Characterization of ICG-pPLGA MPs. (Α) Schematic representation of the preparation process for ICG-pPLGA MPs. (B) SEM images illustrating the morphology of ICG-pPLGA MPs, with a scale bar of 2 μm. Insets provide a magnified view of an individual ICG-pPLGA MP, with a scale bar of 1 μm. (C) Nitrogen sorption isotherms characterizing the porosity of ICG-pPLGA MPs. (D) Hydrodynamic diameter measurements of ICG-pPLGA MPs. Insets include a photographic depiction of ICG-pPLGA MPs. (E) Normalized UV absorption spectra for free ICG, ICG-pPLGA MPs, and ICG-pPLGA MPs. (F) Normalized NIR-I fluorescence spectra of free ICG and ICG-pPLGA MPs, with an excitation wavelength of 730 nm. (G) Normalized NIR-II fluorescence spectra of free ICG and ICG-pPLGA MPs, with an excitation wavelength of 880 nm.

The ICG-pPLGA MPs were synthesized using a modified emulsification-solvent evaporation method (Fig. 1A), with minor modifications to previously reported protocols [48,49]. Clinically used ICG [50] and PLGA [51,52] were selected as the primary materials to ensure the high biocompatibility of the resulting ICG-pPLGA MPs. The nanopores within the PLGA microspheres formed as a result of the decomposition of ammonium bicarbonate, which generated CO₂ and NH₃ gases. These gases induced microsphere rupture, leading to the formation of a porous nanostructure. Finally, gas exchange facilitated the adsorption of clinically used perfluoro propane (C_3_F_8_ gas) into the ICG-pPLGA MPs, enhancing ultrasound contrast. Scanning electron microscopy (SEM) images of the ICG-pPLGA MPs showed a porous, spherical morphology with a uniform size distribution (Fig. 1B). Nitrogen adsorption-desorption curves confirmed the presence of nanostructured pores in the ICG-pPLGA MPs. The average pore size was measured to be 75 nm (Fig. 1C). Dynamic light scattering (DLS) measurements indicated that the ICG-pPLGA MPs had an average particle size of 1.5 ± 0.6 μm (Fig. 1D), smaller than that of clinically used SonoVue microbubbles, which range from 2.0 to 3.0 μm. In contrast, SEM images of the ICG-PLGA MPs showed smooth-surfaced spheres suggesting that the incorporation of ammonium bicarbonate was crucial for the formation of nanopores within the PLGA microspheres (Fig. S1). Spectral analysis revealed a red shift in the maximum absorption peak of the ICG-pPLGA MPs to 803 nm, compared to 780 nm for free ICG (Fig. 1E). The red shift observed in the absorption peak of ICG-pPLGA MPs, compared to free ICG, can be attributed to the encapsulation of ICG molecules within the hydrophobic matrix of p-PLGA MPs. This encapsulation effectively shields ICG from interaction with water and oxygen molecules. ICG-pPLGA MPs have similar maximum emission peak (816 nm) and NIR-II tail peaks as free ICG (Fig. 1F and G). Next, we compared the fluorescence performance of free ICG and ICG pPLGA MPs. We measured the molar extinction coefficient, quantum yields, and fluorescence intensity of them (Fig. S2), and the results showed that ICG doping into pPLGA MPs did not cause significant changes in molar extinction coefficient, NIR-II fluorescence QYs, and NIR-II imaging ability. The results indicate that ICG-pPLGA MPs have significant potential for NIR-II fluorescence imaging.

### 3.2 *In vitro* ultrasound and NIR-II fluorescence of ICG-pPLGA MPs

**Fig. 2:**
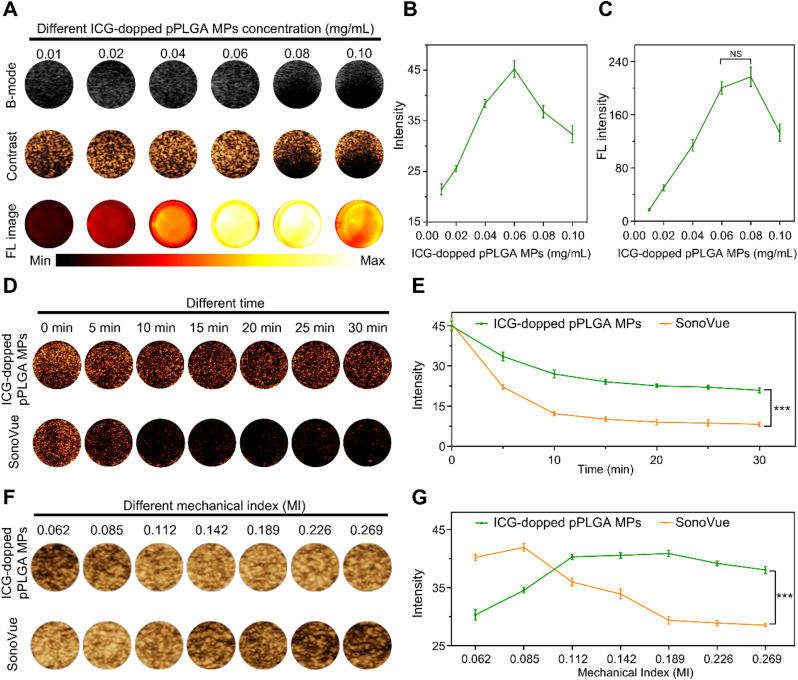
*In vitro* Ultrasound and NIR-II Fluorescence of ICG-pPLGA MPs. (Α) Contrast-enhanced ultrasound images and NIR-II fluorescence images of ICG-pPLGA MPs at varying concentrations (0.01, 0.02, 0.04, 0.06, 0.08, 0.1 mg/mL). (B and C) Quantitative analysis of signal intensity in contrast ultrasound imaging and NIR-II fluorescence imaging for ICG-pPLGA MPs at the specified concentrations. (D) Ultrasound contrast images of ICG-pPLGA MPs and SonoVue at different time intervals (0, 5, 10, 15, 20, 25, 30 min). (E) Quantification of ultrasound signal intensity from D. (F) Ultrasound contrast images comparing ICG-pPLGA MPs and SonoVue under varying mechanical indices (MI) (0.062, 0.085, 0.112, 0.142, 0.189, 0.226, 0.269). (G) Quantification of ultrasound signal intensity from F. NS and ∗∗∗ denote no statistical difference and *P* < 0.001, respectively.

Optimizing ultrasound and NIR-II fluorescence performance of ICG-pPLGA MPs at the adjusted concentration is essential for effective intravital dual-modality imaging. We initially investigated the impact of ICG-pPLGA MP concentration on ultrasound and NIR-II fluorescence imaging performance (Fig. 2A–C). The results demonstrated that the ultrasound contrast signal initially increased with concentration but decreased after reaching a threshold, with optimal contrast achieved at 0.06 mg/mL. At higher concentrations (>0.06 mg/mL), the ultrasound contrast exhibited a "crescent" pattern, suggesting complete absorption of the ultrasound waves by the high concentration of MPs, which prevented wave penetration. Concurrently, the NIR-II fluorescence signal was concentration-dependent, with the optimal fluorescence signal observed at 0.08 mg/mL. Although the fluorescence signal at 0.06 mg/mL was slightly lower than that at 0.08 mg/mL, the 0.06 mg/mL concentration was selected as optimal for both ultrasound and NIR-II fluorescence imaging.

**Fig. 3:**
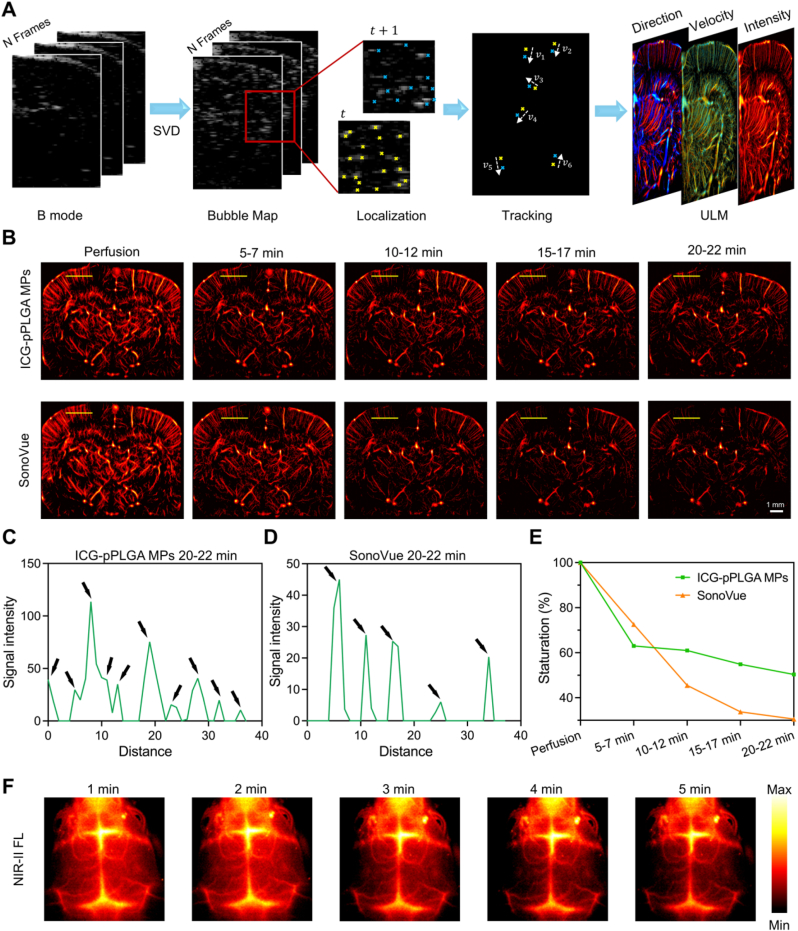
*In vivo* Super-resolution Ultrasound Imaging and NIR-II Fluorescence Dual-Modal Imaging of Cerebrovascular Structures. (Α) Schematic representation of the Super-resolution ultrasound imaging acquisition process and subsequent data processing. (B) Cerebrovascular ULM intensity imaging following the injection of ICG-pPLGA MPs and SonoVue. Scale bar: 1 mm. (C and D) Quantitative maps depicting blood vessel distribution at different time points along the yellow line indicated in Α. ICG-pPLGA MPs at 20–22 min (C) and SonoVue at 20–22 min (D). (E) Temporal changes in image saturation following the injection of ICG-pPLGA MPs and SonoVue. (F) NIR-II imaging of cerebral vasculature post-injection of PMs-ICG. (For interpretation of the references to color in this figure legend, the reader is referred to the Web version of this article.)

Next, we evaluated the ultrasound stability of ICG-pPLGA MPs in comparison to commercially available SonoVue microbubbles. The results revealed that the ultrasound contrast signal decay rate of ICG-pPLGA MPs was slower than that of SonoVue. At the 5-min mark, the contrast signal of ICG-pPLGA MPs was 1.5 times higher than that of SonoVue microbubbles at the same concentration. This suggests that ICG-pPLGA MPs, as solid porous microspheres, exhibit superior ultrasound stability compared to the hollow lipid microbubbles of SonoVue (Fig. 2D and E).

Finally, we assessed the compressive strength of ICG-pPLGA MPs relative to SonoVue microbubbles. At a concentration of 1 × 10⁷/mL, both ICG-pPLGA MPs and SonoVue exhibited an increase in contrast signal intensity with rising mechanical index (MI), which subsequently decreased beyond a certain threshold. The optimal MI for ICG-pPLGA MPs was determined to be 0.189, higher than that of SonoVue microbubbles (0.085) (Fig. 2F and G). These findings highlight that ICG-pPLGA MPs exhibit superior ultrasound and NIR-II imaging performance, along with enhanced stability and pressure resistance compared to SonoVue microbubbles, emphasizing their potential for *in vivo* dual-modality imaging.

### 3.3 *In vivo* super-resolution ultrasound and NIR-II fluorescence imaging

Building on the promising *in vitro* dual-modal imaging capabilities of ICG-pPLGA MPs, we further assessed their *in vivo* performance for cerebrovascular imaging in normal rats. Using a home-built ULM [44], we compared ICG-pPLGA MPs with commercially available SonoVue microbubbles for ultrasound super-resolution imaging of the rat cerebral vasculature. Initially, microbubble data were collected, and the trajectory of each microbubble was tracked to generate maps illustrating velocity, direction, and intensity (Fig. 3Α). Before imaging, the rat's scalp and skull were carefully removed to minimize ultrasound wave attenuation caused by overlying skin and bone tissues. During the first 5 min of perfusion imaging, both the ICG-pPLGA MPs-treated group and the SonoVue-treated group exhibited similar imaging performance, reaching a depth of up to 8 mm (Fig. 3B, Fig. S3). However, as imaging continued, the ICG-pPLGA MPs-treated group revealed a significantly greater number of observable cerebral vessels compared to the SonoVue-treated group (Fig. 3C and D, Fig. S4). Quantitative analysis of microbubble retention showed that 5 min post-intravenous injection, the retention rate of detectable microbubbles was notably higher in the ICG-pPLGA MPs-treated group than in the SonoVue-treated group. Within the 20–22 min imaging window, the retention rate of traceable microbubbles in the ICG-pPLGA MPs-treated group was approximately 50 %, nearly double that of the SonoVue-treated group (Fig. 3E). This enhanced retention is attributed to the superior stability and prolonged *in vivo* persistence of ICG-pPLGA MPs compared to microbubbles.

Subsequently, we assessed the NIR-II fluorescence imaging performance of ICG-pPLGA MPs in a mouse model with an intact scalp and skull. Following intravenous administration of ICG-pPLGA MPs, we observed significant NIR-II fluorescence from the inferior cerebral veins, superior sagittal sinus, transverse sinus, and other cortical veins in both cerebral hemispheres (Fig. 3F). However, NIR-II fluorescence imaging was confined to superficial cerebral vessels beneath the skull, effectively complementing the results obtained from ultrasound super-resolution imaging.

### 3.4 *In vivo* super-resolution ultrasound imaging of ischemic stroke

**Fig. 4:**
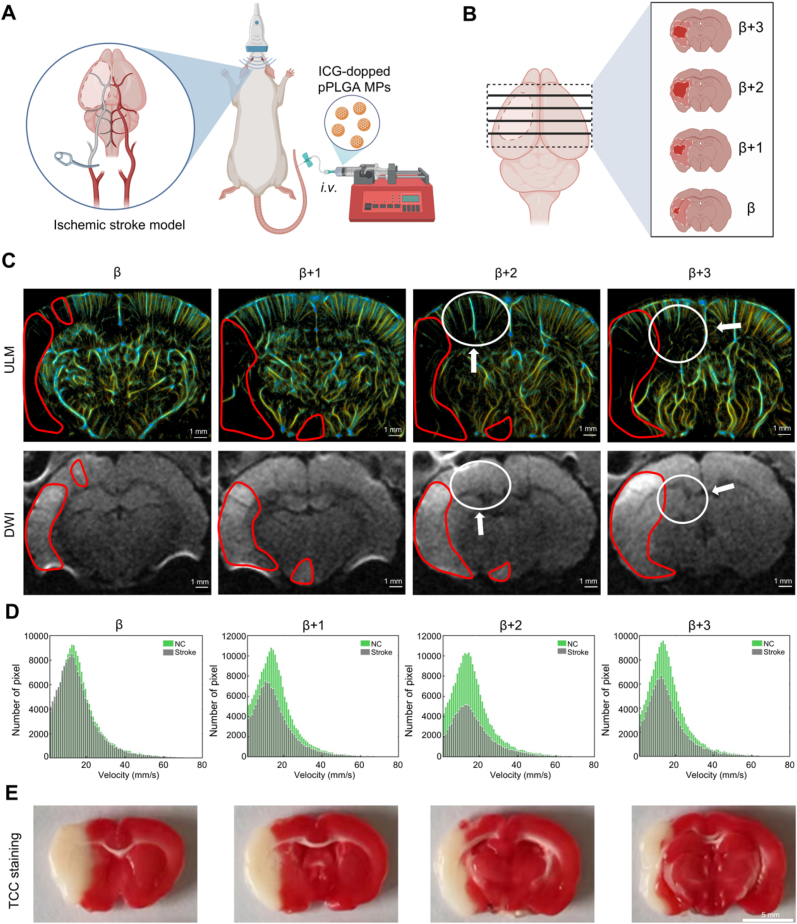
*In vivo* Super-resolution Ultrasound Imaging of Ischemic Stroke. (Α and B) Schematic representation of cerebral microvascular super-resolution ultrasound imaging in an ischemic stroke model. (C) Comparison of super-resolution ultrasound imaging and MRI imaging within the ischemic stroke model. The red region indicates areas where high MRI signals correlate with reduced blood flow observed via ULM. The area circled in white highlights regions where MRI signal elevation is minimal, yet super-resolution ultrasound imaging reveals sparse blood flow signals. Scale bar: 1 mm. (D) Histogram displaying the statistical analysis of microbubble flow velocities. (E) TTC staining of brain slices from a rat model of ischemic stroke. Scale bar: 5 mm. (For interpretation of the references to color in this figure legend, the reader is referred to the Web version of this article.)

We further assessed the super-resolution ultrasound imaging capabilities and diagnostic potential of ICG-pPLGA MPs in a rat model of ischemic stroke. Following the establishment of the ischemic stroke model [46,53], four distinct sagittal brain planes were collected using an *ex vivo* localization system along the rat's longitudinal axis. The initial plane was designated as β = 0, with subsequent planes translated 1 mm toward the bregma, labeled as β+1, β+2, and β+3. ICG-pPLGA MPs were intravenously injected into the rats, and data were continuously collected for approximately 3 min across the four sections (100 data sets per section), followed by the generation of ULM velocity and intensity maps (Fig. 4Α and B, Fig. S5).

MRI images corresponding to the same anatomical positions as the four ultrasound sections were also obtained for comparison. Conventional magnetic resonance diffusion-weighted imaging (DWI) revealed high signal intensity in the right cerebral cortex and parts of the hypothalamus (indicated by red circles), suggesting these regions correspond to the infarcted areas in the model rats. In contrast, super-resolution ultrasound imaging of ICG-pPLGA MPs provided detailed visualization of microvascular structures and their spatial distribution within the infarct zone, achieving a spatial resolution of 25 μm—unattainable with conventional DWI. Furthermore, ULM velocity maps successfully delineated vascular distribution and hemodynamic information in regions inadequately characterized by DWI (indicated by white arrows and circles) (Fig. 4C). These findings demonstrate that super-resolution ultrasound imaging offers superior spatial resolution and enhanced diagnostic sensitivity in the context of cerebral infarction in rat models, surpassing the capabilities of DWI.

We next conducted a quantitative analysis of blood flow velocity in the left and right hemispheres of the brain, measuring blood flow separately in each vascular territory (Fig. S6). The results indicated that blood flow velocity in the infarcted area was significantly lower than that in the normal brain (Fig. 4D). Analysis of ULM intensity maps revealed substantial differences in perfusion areas and the perfusion area ratio between the left and right hemispheres (Table 1). To further validate the infarct regions, TTC staining was performed on rat brain tissue. The staining revealed extensive white areas in the right hemisphere, confirming the presence of infarction, which correlated with findings from both DWI and super-resolution ultrasound imaging (Fig. 4E). The white areas were primarily concentrated in the right cerebral cortex, aligning with low perfusion regions in the ULM images. Notably, TTC staining did not identify ischemic lesions in the hypothalamus, a limitation resolved by super-resolution ultrasound imaging.

**Table 1:** Table of blood flow area and percentage statistics.

Slice		Perfusion Area (mm^2^)	Perfusion Area Ratio (%)
β	NC	19.45	37.39
	Stroke	18.35	35.27
β + 1	NC	23.01	42.38
	Stroke	15.94	28.62
β + 2	NC	22.92	42.29
	Stroke	12.18	21.95
β + 3	NC	19.46	38.73
	Stroke	13.68	26.15

These results highlight that super-resolution ultrasound imaging of ICG-pPLGA MPs not only reveals microstructural and hemodynamic details of the brain vasculature in regions that are insensitive to conventional DWI but also mitigates false negatives associated with TTC staining. This demonstrates the significant potential of super-resolution ultrasound imaging for precise and accurate cerebral imaging.

### 3.5 Biotoxicity of ICG-pPLGA MPs

Low biotoxicity and high biocompatibility are critical for the clinical translation of ICG-pPLGA MPs. We first evaluated the toxicity of ICG-pPLGA MPs at the cellular level, and b End.3 cells were incubated with ICG-pPLGA MPs of different concentrations (0 mg/mL to 5 mg/mL) for 24 h. The results showed that the cell survival rate remained above 90 % at all tested concentrations (Fig. S7), confirming the low cytotoxicity of ICG-pPLGA MP within the detected concentration range.

To systematically evaluate their biotoxicity, ICG-pPLGA MPs were administered intravenously to healthy C57BL/6 mice at a dose twice that of the imaging dose (10 mg/mL). Blood samples were collected 24 h post-injection for complete blood count (CBC) analysis and assessment of liver and kidney function. Seven days later, the mice were euthanized, and tissues from the heart, liver, spleen, lung, and kidneys were harvested for histopathological examination using hematoxylin and eosin (HE) staining.

**Fig. 5:**
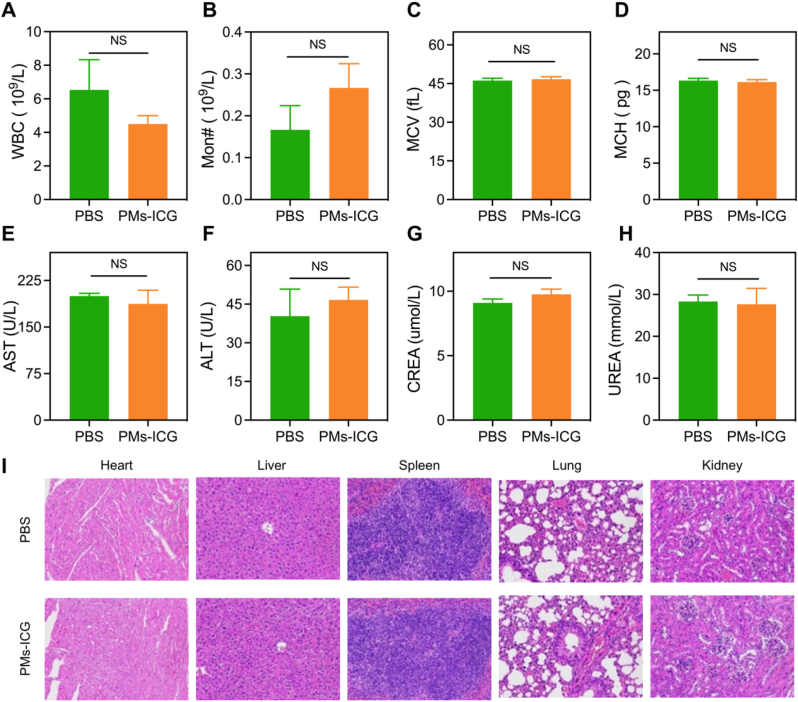
*In vivo* Biotoxicity of ICG-pPLGA MPs. (A to H) Biochemical indicators and routine blood tests for normal mice and those injected with ICG-pPLGA MPs. (WBC: white blood cell; Monocyte: Mon#; MCV: mean corpuscular volume; MCH: mean corpuscular hemoglobin; AST: aspartate transaminase; ALT: alanine aminotransferase; CREA: creatinine). (I) H&E staining of major organs across different experimental groups.

The results indicated that CBC parameters (WBC, MCV, PLT, Mon, MCH), as well as liver function markers (ALT, AST) and renal function indicators (UREA, CREA), remained within normal ranges and showed no significant differences compared to the control group (Fig. 5A–H). Histological analysis revealed no evidence of tissue hemorrhage, necrosis, or inflammation (Fig. 5I). Additionally, *in vitro* hemolysis assays demonstrated that various concentrations of ICG-pPLGA MPs did not induce hemolytic effects in red blood cells (Fig. S8). These findings provide preliminary evidence of the low biotoxicity and high biocompatibility of ICG-pPLGA MPs, supporting their potential for safe clinical application.

Finally, ICG-pPLGA MPs were intravenously administered into BALB/c nude mice, and their biodistribution across various organs was assessed at different time points (10 min, 30 min, 6 h, and 12 h) using near-infrared fluorescence imaging (Fig. S9). The results demonstrated that most ICG-pPLGA MPs accumulated in the liver and lungs, with nearly complete clearance observed by 12 h post-injection, indicating their high biocompatibility and safety profile.

## 4 Conclusions

In this study, we developed ICG-pPLGA MPs for dual-modal imaging of ischemic stroke, combining super-resolution ultrasound imaging and NIR-II fluorescence imaging. Our findings demonstrate that the rigid structure of ICG-pPLGA MPs enhances stability in *in vivo* circulation, enabling prolonged imaging sessions without the need for continuous injections. This dual-modal approach provides comprehensive visualization of both superficial and deep cerebral vasculature, addressing the limitations of individual imaging techniques. *In vivo* experiments in a rat model of ischemic stroke showed that ICG-pPLGA MPs significantly improved super-resolution ultrasound imaging performance, allowing for detailed visualization of microvascular architecture and hemodynamics. This approach achieved higher spatial resolution than traditional DWI, offering critical insights into infarct regions that are often poorly characterized. Biotoxicity assessments confirmed the low toxicity and high biocompatibility of ICG-pPLGA MPs, supporting their favorable safety profile for potential clinical applications. These findings highlight the potential of integrating super-resolution ultrasound and NIR-II fluorescence imaging with ICG-pPLGA MPs for more accurate detection of ischemic stroke, paving the way for enhanced diagnostic methodologies.

## CRediT authorship contribution statement

**Ziyue Li:** Writing – original draft, Validation, Investigation. **Yu Qiang:** Data curation. **Dongli Chen:** Validation, Investigation. **Dehong Hu:** Supervision, Funding acquisition. **Duyang Gao:** Supervision, Conceptualization. **Xiaohua Xu:** Supervision, Conceptualization. **Lei Sun:** Supervision, Conceptualization. **Yingjia Li:** Writing – review & editing, Funding acquisition. **Weibao Qiu:** Writing – review & editing, Funding acquisition. **Zonghai Sheng:** Writing – review & editing, Funding acquisition.

## Ethics approval and consent to participate

All the animal protocols were approved by Ethical Committee of the Shenzhen Institute of Advanced Technology, Chinese Academy of Sciences (approval number: SIAT-IACUC-220921-YGS-SZH-A2188) and also in accordance with the policy of the National Institute of Health (China).

## Consent for publication

All authors agree for publication. Part of the schematic images were created with BioRender.com, with permission.

## Declaration of competing interest

The authors declare that they have no known competing financial interests or personal relationships that could have appeared to influence the work reported in this paper.

The author is an Editorial Board Member/Editor-in-Chief/Associate Editor/Guest Editor for this journal and was not involved in the editorial review or the decision to publish this article.

## Data Availability

Data will be made available on request.
