# Supplementary material for: Dual-modal super-resolution ultrasound and NIR-II fluorescence imaging of ischemic stroke with ICG-doped porous PLGA microspheres
Source: Mater Today Bio. 2025 Jan 22;31:101513. doi: 10.1016/j.mtbio.2025.101513 (PMC11803314; doi:10.1016/j.mtbio.2025.101513)
Supplement: Multimedia component 1 [file mmc1.docx]

**Supplementary Information**

**Dual-Modal Super-Resolution Ultrasound and NIR-II Fluorescence Imaging of Ischemic Stroke with ICG-doped Porous PLGA Microspheres**

Ziyue Li^a,b†^, Yu Qiang^b,c†^, Dongli Chen^b,d†^, Dehong Hu^b^, Duyang Gao^b^, Xiaohua Xu^d^, Lei Sun^c^, Yingjia Li*^a^, Weibao Qiu*^b^, Zonghai Sheng*^b^

a. Department of Medicine Ultrasonics, Nanfang Hospital, Southern Medical University, Guangzhou 510515, China.

b. Research Center for Advanced Detection Materials and Medical Imaging Devices, Paul C. Lauterbur Research Center for Biomedical Imaging, Institute of Biomedical and Health Engineering, Shenzhen Institute of Advanced Technology, Chinese Academy of Sciences, Shenzhen 518055, China.

c. The Hong Kong Polytechnic University, Department of Biomedical Engineering, Hong Kong 999077, China.

d. Division of Ultrasound, The University of Hong Kong-Shenzhen Hospital, No.1, Haiyuan Road, Futian District, Shenzhen 518053, China.

†Ziyue Li, Yu Qiang, Dongli Chen contributed equally to this work.

* Corresponding authors.

*Email addresses*: lyjia@smu.edu.cn (Y. Li), wb. qiu@siat.ac.cn (W. Qiu) and zh.sheng@siat.ac.cn (Z. Sheng).


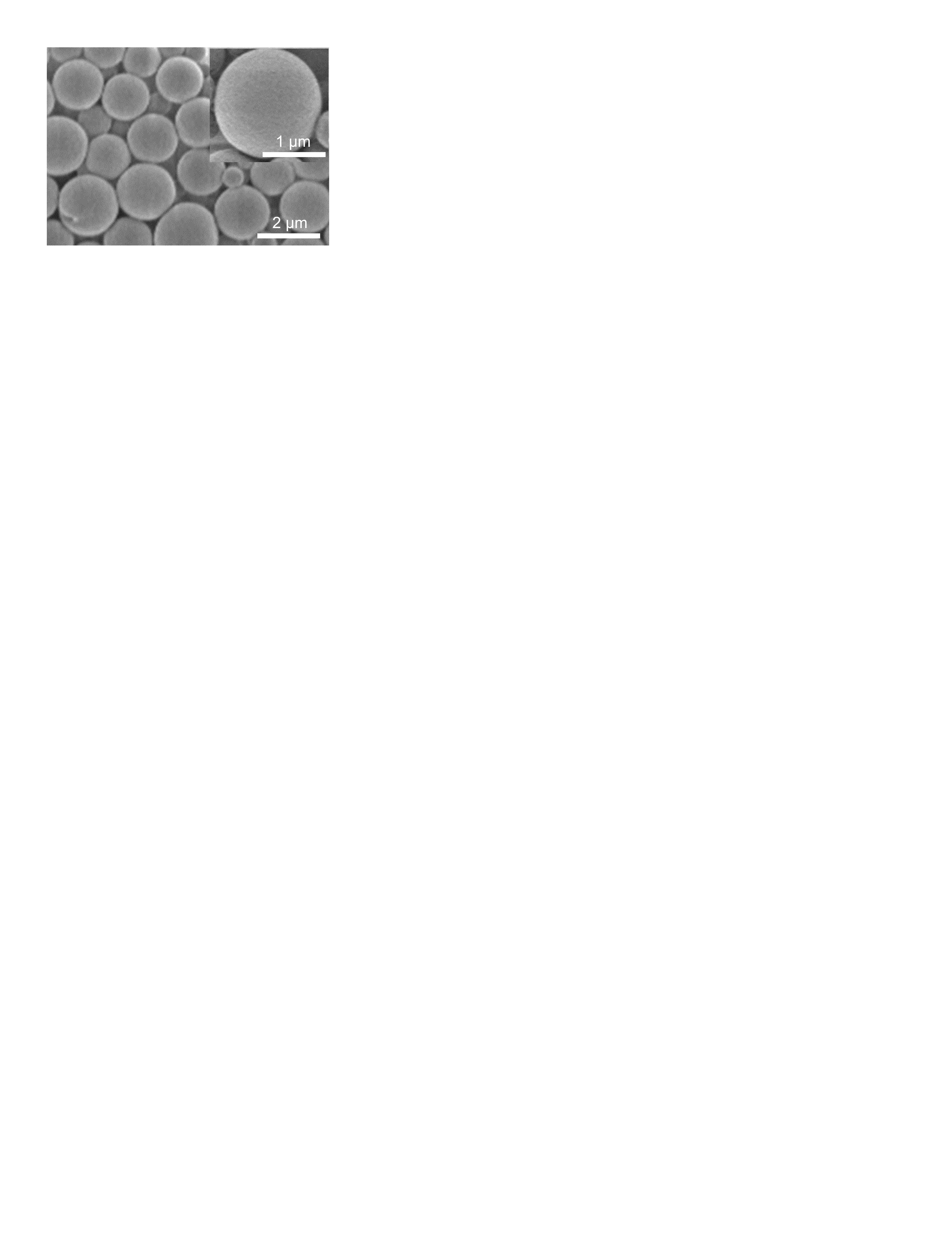


**Figure S1.** SEM images of ICG-PLGA MPs. The scale bar represents 2 μm. Inset shows a magnified view of a single ICG-PLGA MP, with a scale bar of 1 μm.


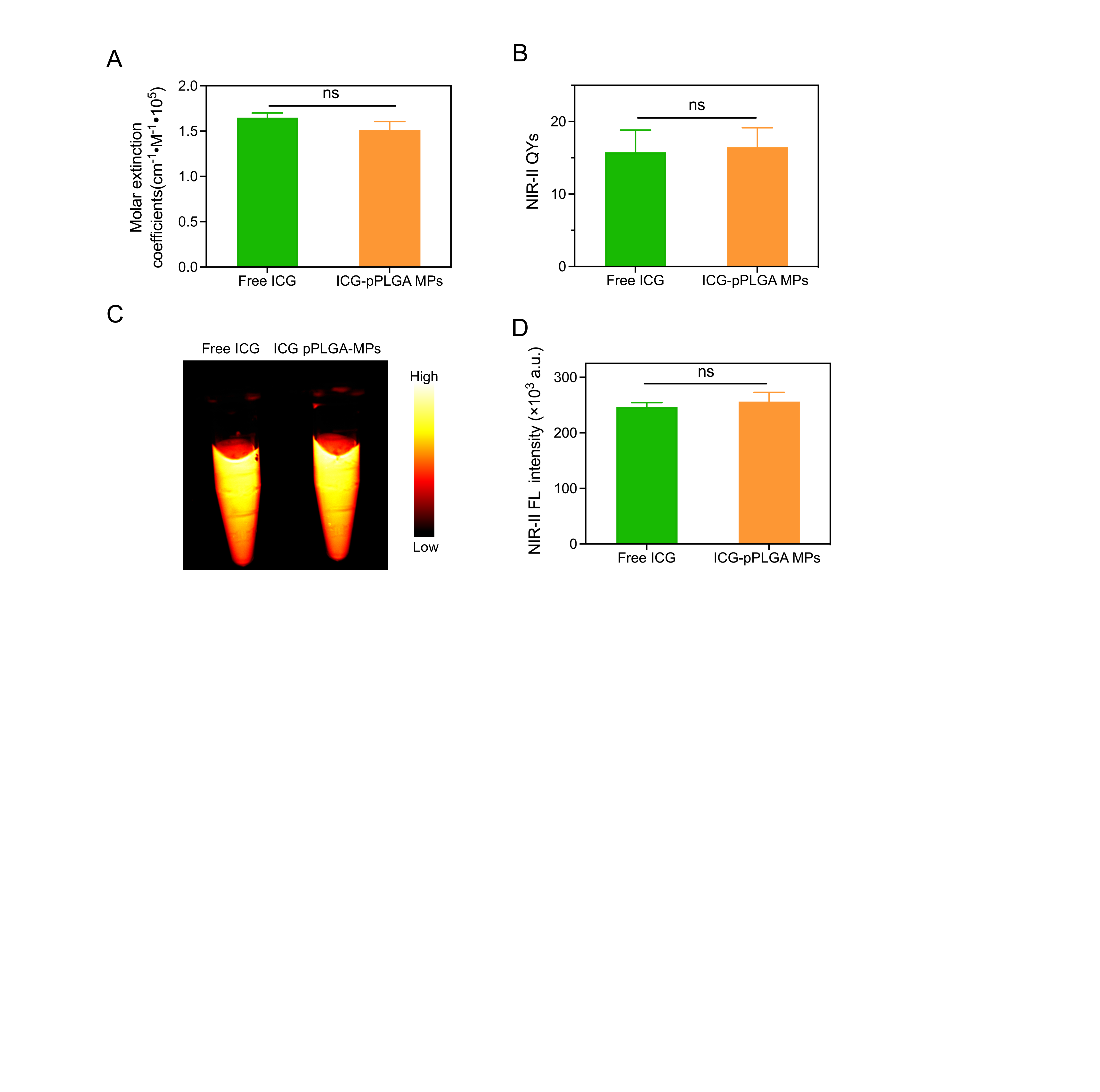


**Figure S2.** (A) Molar extinction coefficients of at ICG and ICG-pPLGA MPs at 780 nm (n = 3; mean ± SD). (B) The NIR-II QYs comparison of free ICG and ICG-pPLGA MPs (n = 3; mean ± SD). (C) NIR-II fluorescence images of free ICG and ICG-pPLGA MPs, ICG concentration: 5 µg mL⁻¹, excitation wavelength: 808 nm, 1000 nm long-pass filter, exposure time: 10 ms. (D) Quantification of NIR-II fluorescence images (n = 3; mean ± SD). ns, denote no statistical difference.


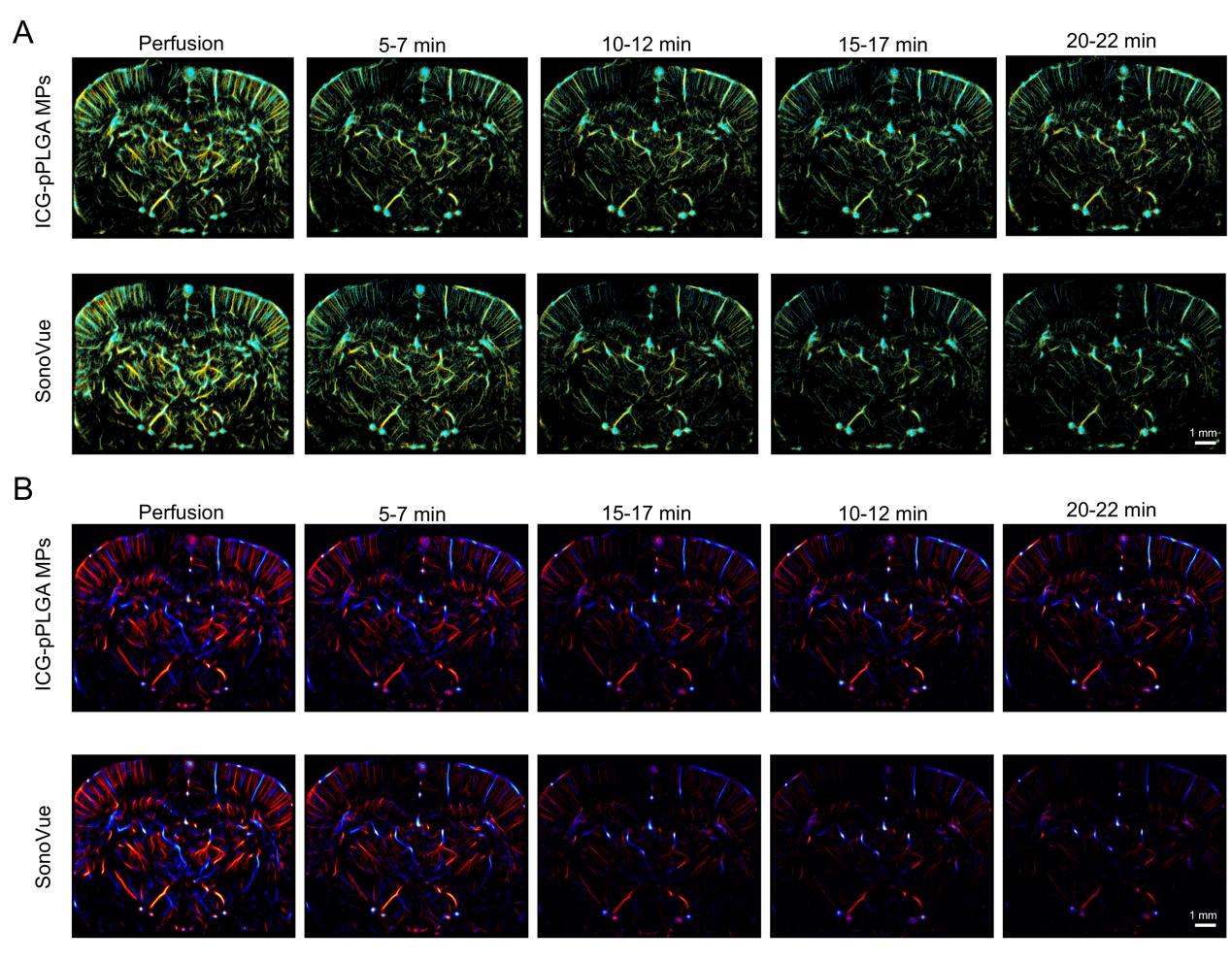


**Figure S3.** Super-resolution ultrasound cerebrovascular imaging. (A) Cerebrovascular ULM velocity imaging following the intravenous injection of ICG-pPLGA MPs (4 × 10^8^/mL) and SonoVue (4 × 10^8^/mL), respectively. Scale bar: 1 mm. (B) Cerebrovascular ULM directional imaging after intravenous injection of ICG-pPLGA MPs and SonoVue, respectively. Scale bar: 1 mm.


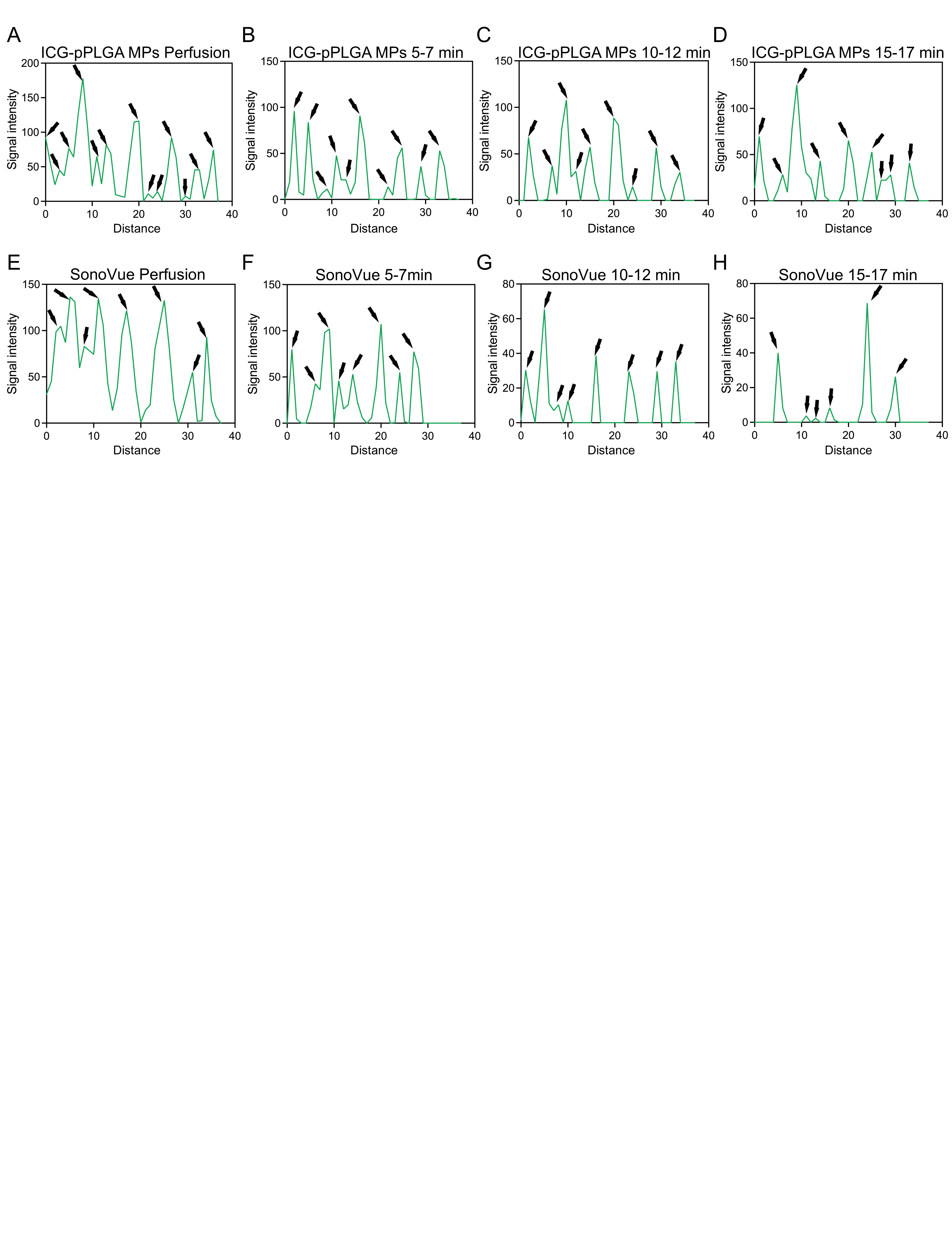


**Figure S4.** (A to D) Quantitative analysis of the number of blood vessels using ULM imaging at different time points after intravenous injection of ICG-pPLGA MPs. (E to H) Quantitative analysis of the number of blood vessels using ULM imaging at different time points after intravenous injection of SonoVue microbubbles.


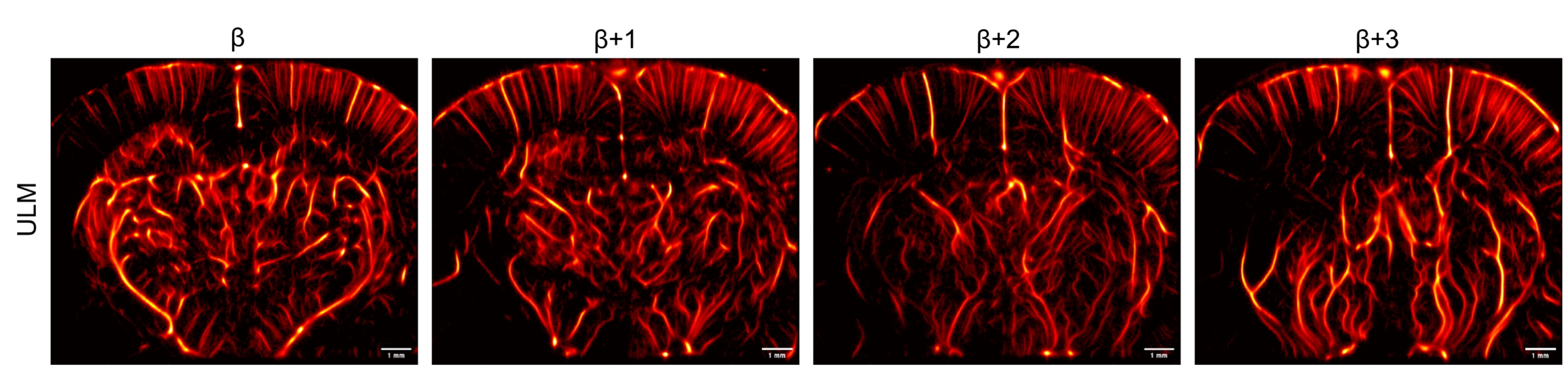


**Figure S5.** Representative intensity maps reconstructed from ULM imaging depict vascular distribution at different brain planes (β, β+1, β+2, β+3) of ischemic stroke model. Scale bar = 1 mm.


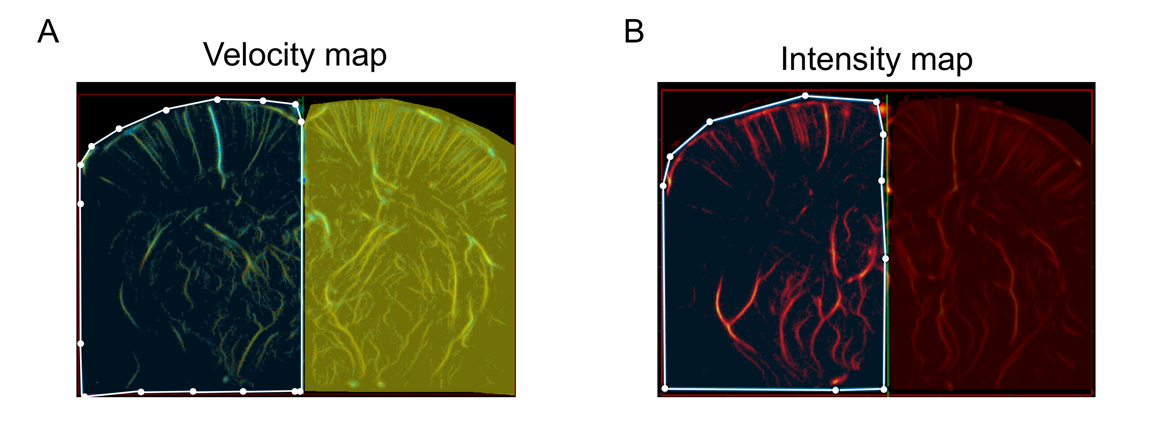


**Figure S6.** The method of boundary division for analysis of cerebrovascular blood flow velocity (A) and intensity (B) in the left and right cerebrum, respectively. The white solid line area represents the hemisphere where a stroke occurs.


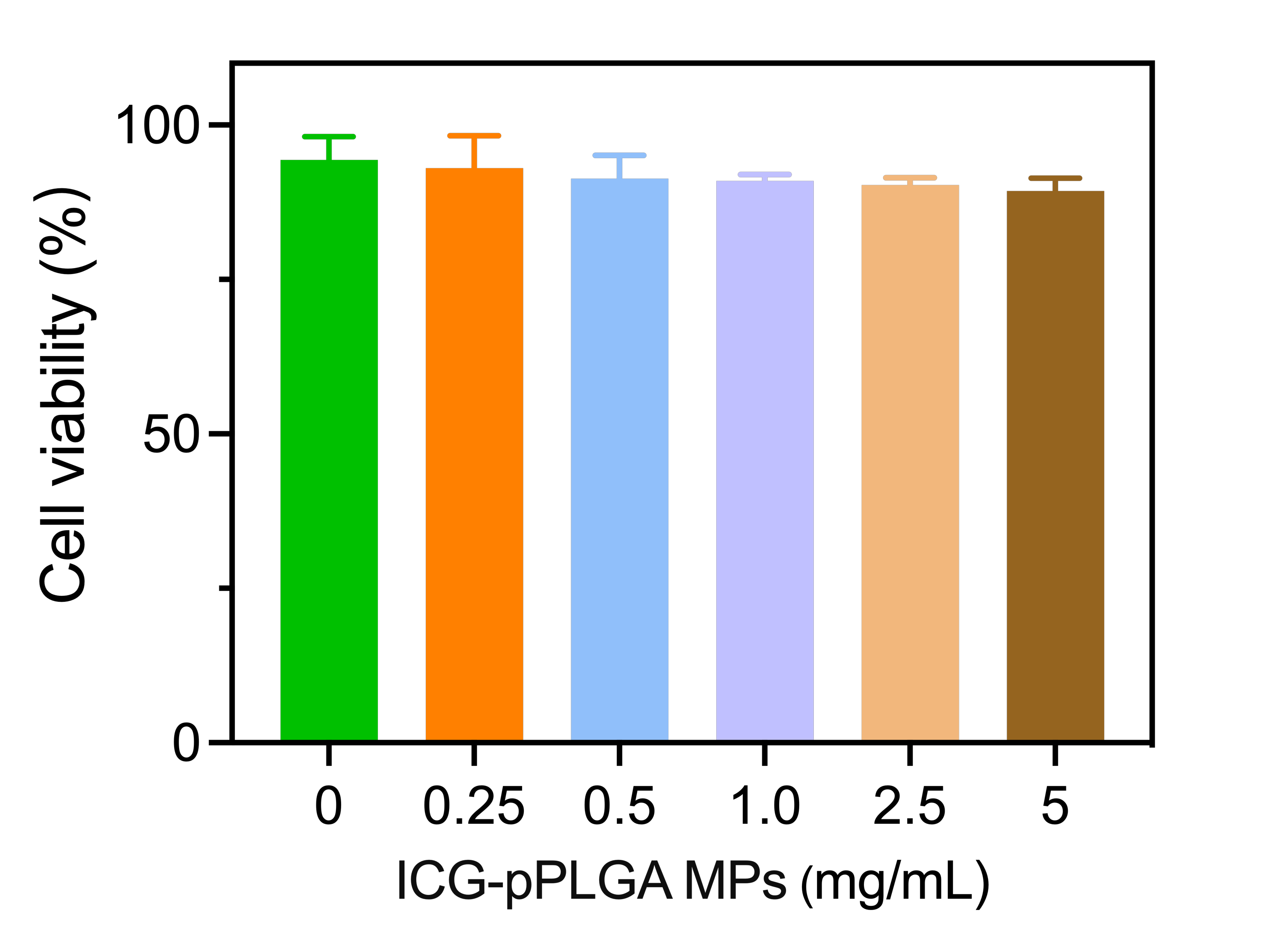


**Figure S7.** The cytotoxicity of ICG-pPLGA MPs against b. End3 cells after incubation for 24 hours (n = 5; mean ± SD).


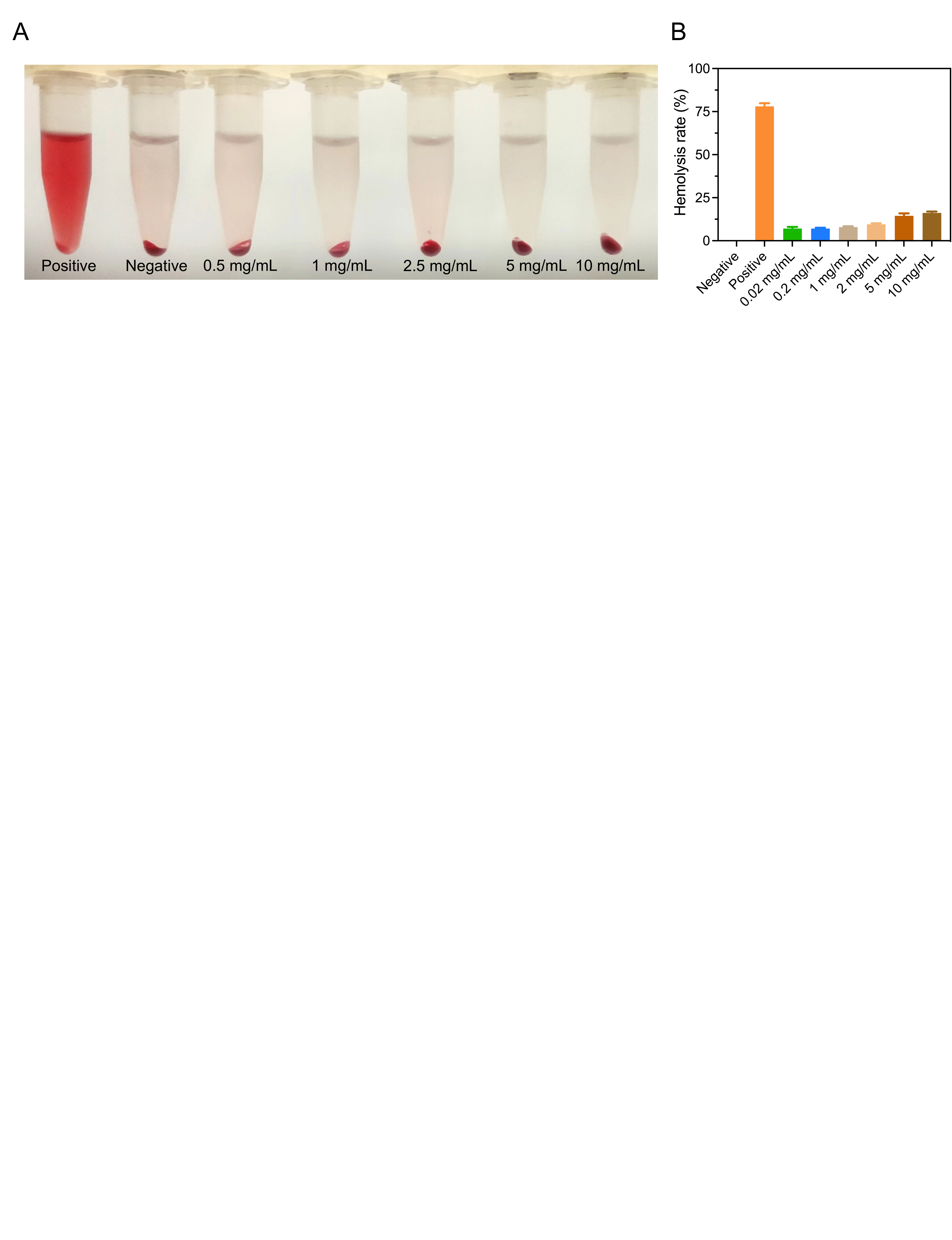


**Figure S8.** (A) Photographs and (B) hemolysis assay of negative, positive and ICG-pPLGA MPs with different concentrations (n = 3; mean ± SD).


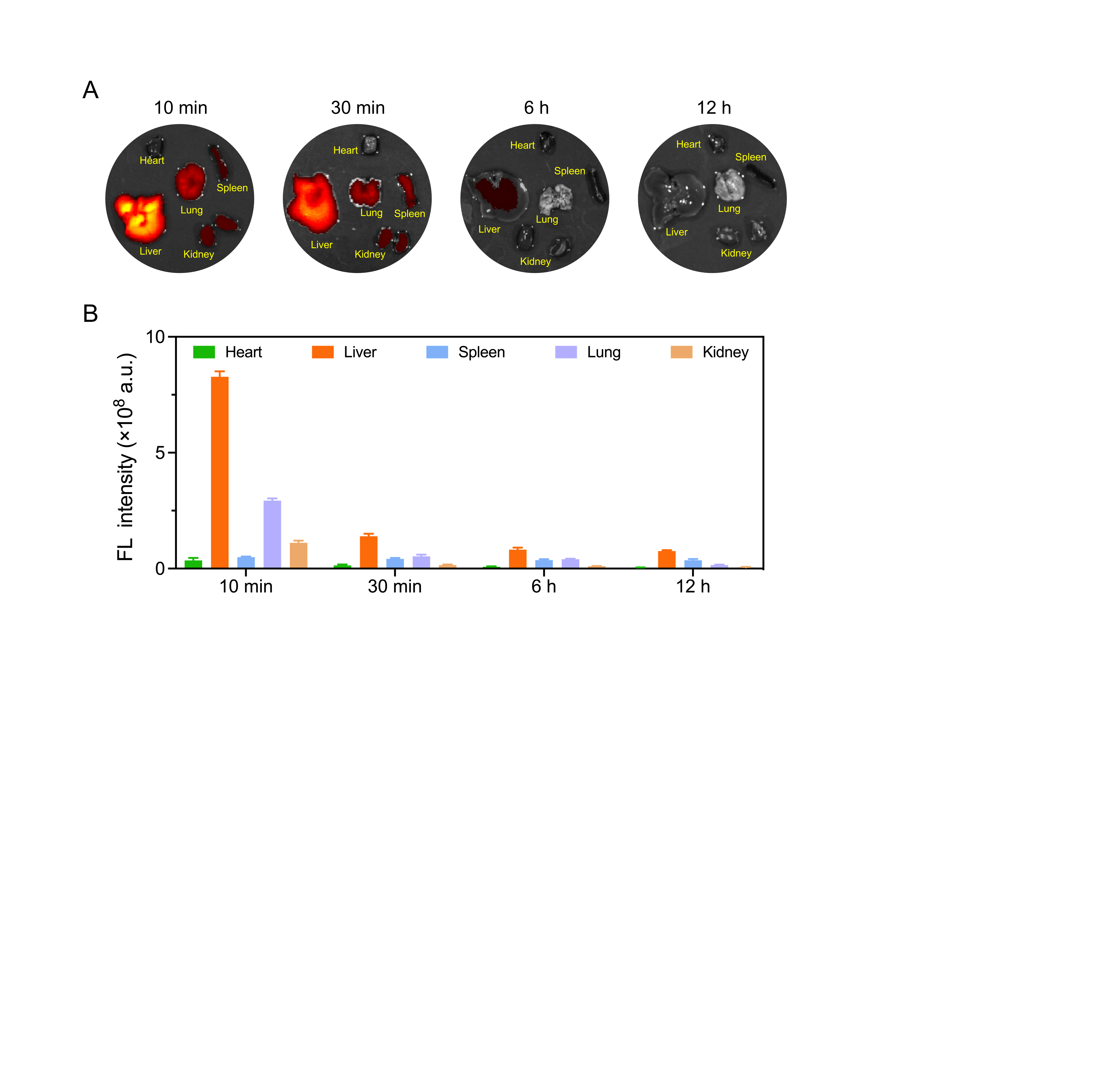


**Figure S9.** (A) Distribution of major organs at different times after injection of ICG-pPLGA MPs. (B) Quantitative analysis of fluorescence intensity of major organs (n = 3; mean ± SD).
